# Cardiogenic Shock Due to Multisystem Inflammatory Syndrome in a Young Adult Female

**DOI:** 10.7759/cureus.16380

**Published:** 2021-07-14

**Authors:** Amer Muhyieddeen, Mehdia Amini, Damon McEnroe, Felice Lin

**Affiliations:** 1 Medicine, University of California San Francisco (UCSF) Fresno, Fresno, USA; 2 Internal Medicine, University of California San Francisco (UCSF) Fresno, Fresno, USA; 3 Cardiology, University of California San Francisco (UCSF) Fresno, Fresno, USA

**Keywords:** acute cardiogenic pulmonary edema, shock, kawasaki disease shock syndromes, cardio, cardio toxicity, covid, covid 19, multisystem inflammatory syndrome in adult

## Abstract

Multisystem inflammatory syndrome in adults (MIS-A) was initially described by pediatricians after reporting a temporal association of a mimicker of Kawasaki disease shortly after the resolution of a COVID-19 illness. Since June 2020, there have been an increased amount of reports of adults and adolescents above the age of 18 presenting with the syndrome. We report a case of a 20-year-old female with no medical history who presented with cardiogenic shock and was found to have MIS-A.

## Introduction

We report a case of a young 20-year-old female with no significant medical, family, and social history who presented to our hospital in cardiogenic shock. The patient had recently recovered from a severe acute respiratory syndrome coronavirus 2 (SARS-CoV-2) infection two months prior, where her only complaint was cough. During her prior SARS-CoV-2 infection, her oxygen saturation and vitals were normal, and she did not require treatment with steroids or other medical therapy. All infectious workup were negative, and the patient was not pregnant. A cardiac magnetic resonance imaging (CMR) was ordered to evaluate for infiltrative causes of heart failure. CMR revealed myocardial edema predominantly in the entire septum with a reduced left ventricular ejection fraction (LVEF). The presence of a reduced LVEF, myocardial edema in the absence of necrosis, and pericardial effusion were highly suggestive of acute/subacute myocarditis. The patient was started on dexamethasone IV 6 mg for 10 days, followed by one month taper on discharge. Since June 2020, there have been 49 reported multisystem inflammatory syndrome (MIS) cases in adults and adolescents above the age of 18 [1]. Though little is known about the syndrome, it is suspected to be a post-infectious syndrome rather than an acute one. The vast majority of MIS cases have been described in children, but cases have been described increasingly in adults. Clinicians should suspect multisystem inflammatory syndrome in adults (MIS-A) in patients who have recently recovered from an acute COVID-19 illness that presents with symptoms of cardiogenic shock in the absence of respiratory compromise. It is important to identify these patients early to avoid the possible morbidity associated with it.

## Case presentation

A 20-year-old female presented to the emergency department with a complaint of subjective fevers, intermittent chest pain, nausea, vomiting, and a new diffuse morbilliform rash. The patient had recently recovered from a SARS-CoV-2 infection two months prior, where her only complaint was cough and she had no other post-COVID-19 syndrome symptoms. On physical examination, the patient was febrile at 39.6 C, hypotensive with a blood pressure of 80/50, and tachycardic with a heart rate above 130. The patient had no respiratory symptoms, and oxygen saturation was normal. The patient was noted to have elevated jugular venous distension, normal rhythm with the absence of any murmur, normal breath sounds on bilateral lung fields, a diffuse morbilliform rash on her left arm, and no peripheral lower-extremity edema.

**Figure 1:**
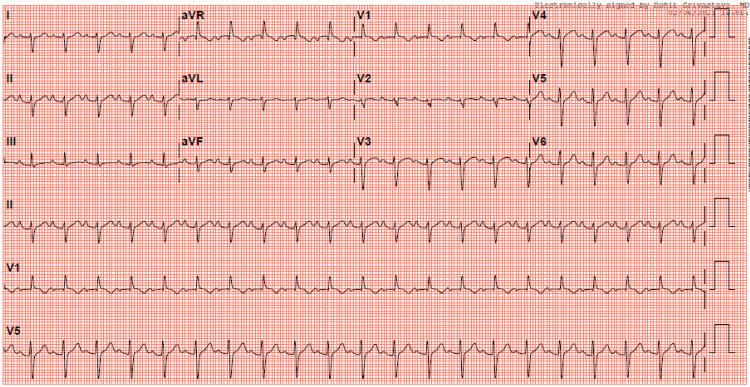
Sinus tachycardia with an extreme right axis deviation and evidence of right ventricular hypertrophy.

**Figure 2:**
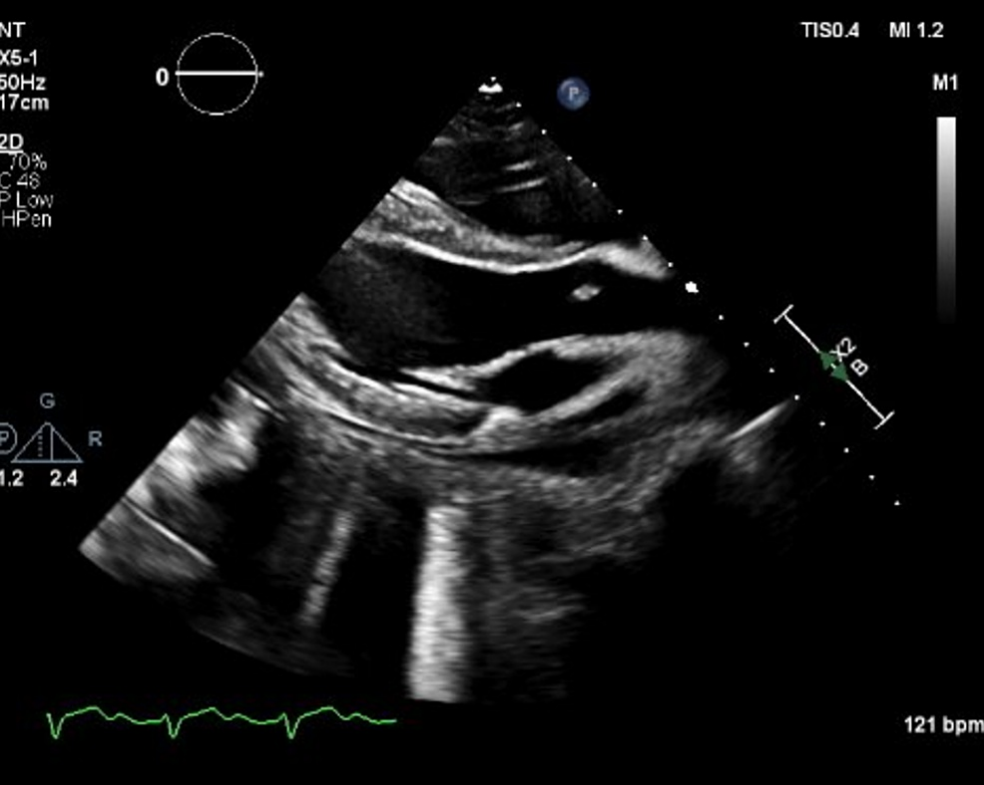
Transthoracic echocardiogram.

**Figure 3:**
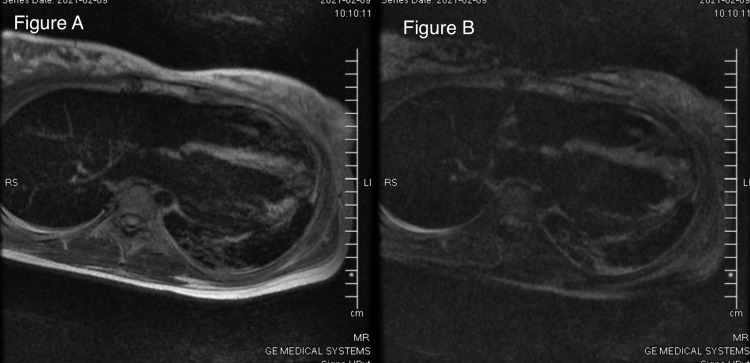
(A) Shows increased T2 signal in double inversion recovery sequence. (B) Shows increased T2 signal in triple inversion recovery sequence.

An electrocardiogram showed sinus tachycardia with an extreme right axis deviation and evidence of right ventricular hypertrophy (Figure 1). Laboratory workup was notable for leukocytosis of 25,700 per uL, elevated brain natriuretic peptide of 1757 pg/mL, elevated troponin of 1.270 ng/mL, and high inflammatory markers (erythrocyte sedimentation rate 106 mm and D-dimer 3369 ng/mL). Chest X-ray and CT angiogram were unremarkable. The patient was admitted to the medical intensive care unit for hemodynamic monitoring and vasopressor support. Blood cultures and remaining infectious workup for bacterial and viral causes were negative. Repeat SARS-CoV-2 polymerase chain reaction from the nasopharyngeal swab was negative. A swan catheter was placed to determine the etiology of her shock. On placement of swan catheter, her hemodynamic data while on 5 mcg/min of norepinephrine revealed a right atrial pressure of 8 mmHg, pulmonary artery pressure of 25/15 mmHg, and a pulmonary capillary wedge pressure of 16. Her cardiac output per Fick’s equation was 6.7 L/min, and her cardiac index was 4.0 L/min/m^2^. An echocardiogram was performed and it showed normal left ventricle (LV) chamber size and moderately decreased systolic function with an estimated ejection fraction of 35-40% (Figure 2). The right ventricle chamber size was normal with mildly reduced systolic function. A CMR image was ordered to evaluate for infiltrative causes of heart failure. CMR revealed increased T2 signal in double inversion recovery and triple inversion recovery sequences consistent with myocardial edema seen predominantly in the entire septum (Figure 3). The LV was noted to be mildly dilated with a reduced LVEF. The presence of a small pericardial effusion was also noted without evidence of pericardial enhancement. The presence of a reduced LVEF, myocardial edema in the absence of necrosis, and pericardial effusion were highly suggestive of acute/subacute myocarditis.

 

Given that the patient was in septic shock on presentation to the emergency department, she was adequately fluid resuscitated and subsequently started on vasopressor support. Cardiology was consulted, and given the clinical and imaging findings, the patient was highly suspected to have MIS-A. Due to the high risk of post-MIS complications such as aortitis and coronary aneurysms, the patient was started on Decadron IV 6 mg for 10 days followed by one-month taper on discharge. The patient was able to be weaned off vasopressor support within three days and was eventually discharged in a normal state of health. Prior to discharge, a CT angiogram of coronary arteries was ordered and it showed no atherosclerosis, aneurysms, and significant aortic inflammatory changes (Video 1).

**Video 1:** Normal CT coronary artery angiogram without any atherosclerosis or aneurysms, and without any significant aortic inflammatory changes.

## Discussion

SARS-CoV-2 first appeared in Wuhan, China, at the end of 2019 and rapidly spread across the globe within a few months. The first reports from China showed that pediatric patients tended to present with milder symptoms of the virus when compared to adults [2]. However, this has recently been challenged by the emergence of children appearing with a severe hyperinflammatory syndrome very similar to Kawasaki disease. MIS was initially described by pediatricians after reporting a temporal association of a mimicker of Kawasaki disease shortly after the resolution of a COVID-19 illness [3]. Since June 2020, there have been 49 reported cases of MIS in adults and adolescents above the age of 18 [1]. Though little is known about the syndrome, it is suspected to be a post-infectious syndrome rather than an acute one. The vast majority of MIS cases have been described in children but cases have been described increasingly in adults as well.

The characteristic symptoms used to diagnose MIS are extensive and are based on Centers for Disease Control and Prevention's recommendations. This includes the presence of fever with an associated increase in inflammatory biomarkers, laboratory confirmation of a previous COVID-19 infection within 12 weeks of symptom onset, and lack of a severe respiratory illness. In addition, at least two of the subsequent criteria must be met: clinical evidence of mucocutaneous inflammatory changes such as rash and conjunctivitis, low blood pressure leading to shock, and evidence of cardiac involvement. Cardiac involvement can range from the evidence of myocarditis, pericarditis, new echographic abnormalities, and clinical symptoms of heart failure [4]. Finally, the presence of new-onset gastrointestinal symptoms such as abdominal pain, vomiting, and diarrhea should be present [5]. Our patient presented with all the major aforementioned symptoms, thus allowing us to confidently make our diagnosis.

Currently, there exists no widely accepted guideline for the treatment of MIS-A. The American College of Rheumatology has published a diagnosis and treatment guide for MIS in children (MIS-C) [6]. Treatment modalities for MIS-A have been deduced from the suggested therapies for MIS-C as both conditions are indistinguishable. Generally, this involves supportive care and therapy against the underlying inflammatory process with IV immunoglobulin and steroid therapy [7].

It is important to identify cardiac involvement in MIS-A, which includes myocardial fibrosis, edema, and pericarditis as it is associated with a worse prognosis [8]. CMR imaging is the gold standard in evaluating cardiac morphology and function and is the ideal imaging modality to evaluate patients with MIS. In contrast to other imaging modalities, CMR not only targets functional and morphological abnormalities but tissue pathology as well. It is possible to quantifiably assess myocardial fibrosis and edema through CMR mapping techniques such as T1, T2, and extracellular volume. In a recent study by Huang et al., which involved 26 patients who had recovered from COVID-19 but had cardiac symptoms, 15 of the 26 patients showed myocardial edema and/or foci late gadolinium enhancement (LGE) [9]. Myocardial edema and foci LGE lesions were the prominent CMR image manifestations seen in this cohort. Usually, acute viral myocarditis causes edema to be visible via T2 signal hyperintensity in the inferior and inferior-lateral walls of the ventricle [10]. However, the majority of T2 hyperintensity in SARS-CoV-2-associated myocarditis appeared in the interventricular septum, and anterior, antero-lateral, and inferior walls [9]. Interestingly, on our patient’s CMR, diffuse myocardial edema was most prominent in the interventricular septum.

## Conclusions

Clinicians should suspect MIS-A in patients who have recently recovered from an acute COVID-19 illness that presents with symptoms of cardiogenic shock in the absence of respiratory compromise. It is important to identify these patients early to avoid the possible morbidity associated with it. CMR is an attractive option for a detailed evaluation due to non-invasiveness, lack of radiation, and excellent image resolution.
